# Measuring costs of data collection at village clinics by village doctors for a syndromic surveillance system-a cross sectional survey from China

**DOI:** 10.1186/s12913-015-0965-2

**Published:** 2015-07-25

**Authors:** Yan Ding, Yang Fei, Biao Xu, Jun Yang, Weirong Yan, Vinod K. Diwan, Rainer Sauerborn, Hengjin Dong

**Affiliations:** Institute of Public Health, Heidelberg University, Heidelberg, Germany; School of Public Health, Fudan University, Shanghai, China; Yongxiu County center for Disease Control and Prevention, Yongxiu, China; Tongji Medical School, Huazhong University of Science and Technology, Wuhan, China; Institute for Global Health, Karolinska Institutet, Stockholm, Sweden; Center for Health Policy Studies, Zhejiang University School of Medicine, Hangzhou, China

**Keywords:** Rural China, Village clinics, Infectious diseases, Syndromic surveillance system, Data collection, Staff time costs, Depreciation and opportunity costs

## Abstract

**Background:**

Studies into the costs of syndromic surveillance systems are rare, especially for estimating the direct costs involved in implementing and maintaining these systems. An Integrated Surveillance System in rural China (ISSC project), with the aim of providing an early warning system for outbreaks, was implemented; village clinics were the main surveillance units. Village doctors expressed their willingness to join in the surveillance if a proper subsidy was provided. This study aims to measure the costs of data collection by village clinics to provide a reference regarding the subsidy level required for village clinics to participate in data collection.

**Methods:**

We conducted a cross-sectional survey with a village clinic questionnaire and a staff questionnaire using a purposive sampling strategy. We tracked reported events using the ISSC internal database. Cost data included staff time, and the annual depreciation and opportunity costs of computers. We measured the village doctors’ time costs for data collection by multiplying the number of full time employment equivalents devoted to the surveillance by the village doctors’ annual salaries and benefits, which equaled their net incomes. We estimated the depreciation and opportunity costs of computers by calculating the equivalent annual computer cost and then allocating this to the surveillance based on the percentage usage.

**Results:**

The estimated total annual cost of collecting data was 1,423 Chinese Renminbi (RMB) in 2012 (P25 = 857, P75 = 3284), including 1,250 RMB (P25 = 656, P75 = 3000) staff time costs and 134 RMB (P25 = 101, P75 = 335) depreciation and opportunity costs of computers.

**Conclusions:**

The total costs of collecting data from the village clinics for the syndromic surveillance system was calculated to be low compared with the individual net income in County A.

## Background

Studies of the costs of syndromic surveillance systems are rare, especially for estimating the direct costs involved in implementing and maintaining these systems. There was only one study before 2014 [1] elucidating the direct costs of implementing and maintaining such a system. This study [1] excluded the costs incurred during the process of data collection at hospitals because those hospitals routinely collected the data elements in their electronic information system; however, some other syndromic surveillance systems may require substantial resources to be devoted to data collection. In these cases, the costs of data collection should be explored in order to arrive at comprehensive costs for implementing and maintaining these surveillance systems.

An Integrated Surveillance System in rural China (ISSC project), which integrated the functions of a syndromic surveillance system into the existing infectious disease surveillance system in China, was established and implemented from April 2012 to March 2014 with village clinics as the major surveillance units [2]. The aim of this project was to improve early warning for infectious disease outbreaks in rural China and it required additional effort in data collection at village clinics. Data for the surveillance at those clinics was collected from outpatients regarding 10 non-specific symptoms, and those symptoms were fever, cough, sore throat, nausea/vomiting, diarrhea, rash, muco-cutaneous hemorrhage, headache, convulsion and disturbance of consciousness [2]. The village clinics did not have electronic medical records that could be sorted and analyzed for surveillance purposes. Instead the village doctors entered data daily specifically for the syndromic surveillance to the ISSC platform. Our hypothesis was that this surveillance was labor intensive and that the costs of data collection for the implementation of the surveillance would not be negligible for those village clinics.

In a previous study, we found that the village doctors would be willing to provide public health services, including syndromic surveillance services, if they received an appropriate subsidy [3]. The purpose of this paper is to measure the costs of data collection by village clinics for the syndromic surveillance system in the ISSC project, and to provide a reference regarding the subsidy level required for village clinics to participate in data collection. The costs reported here included the time costs of data collection, and the depreciation and opportunity costs of the village clinic computers.

### Definitions

According to the CDC’s evaluation framework, syndromic surveillance for early outbreak detection is defined as “an investigational approach, where health department staff, assisted by automated data acquisition and generation of statistical signals, monitor disease indicators continually (real-time) or at least daily (near real-time) to detect outbreaks of diseases earlier and more completely than might otherwise be possible with traditional public health methods” [4]. In the ISSC project, the syndromic surveillance system was an investigational approach, where staff from the County CDC, as well as researchers in this project, assisted by automated data acquisition and generation of statistical signals, monitored syndromes daily based on the 10-symptom data reported by the village clinics to detect outbreaks of diseases earlier and more comprehensively than might otherwise be possible with the existing infectious disease surveillance system in China. The existing system in China is a notifiable case report system based on confirmed infectious diseases [5]. Diseases of concern in this syndromic surveillance system were acute respiratory infectious diseases (e.g., influenza, epidemic cerebrospinal meningitis, measles, chicken pox, mumps), as well as gastrointestinal infectious diseases (e.g., bacillary dysentery, enteritis, viral hepatitis, polio) [6]. The syndromic surveillance system also detected outbreaks based on absenteeism data from primary schools and sales of medicines from pharmacies, which is beyond the scope of this survey. More information can be found in other studies [2, 7].

Any report concerning an outpatient with at least one of the ten symptoms became a reported event to the syndromic surveillance system. Syndromes defined within this system included acute respiratory infection (patients with fever and either cough or sore throat), influenza-like illness (patients with temperature ≥38 °C and with either cough or sore throat), fever gastro syndrome (patients with fever and with either diarrhea or nausea/vomiting), and fever and rash (patients with both fever and rash) [8].

## Methods

This cost analysis was performed from the program managers’ point of view. The time horizon of this study was 15 months, covering the formal implementation period of ISSC in County A from April 1, 2012, to June 30, 2013. All cost data were reported in Chinese currency (Renminbi; RMB), and costs were not discounted or adjusted for inflation due to the short time horizon.

### Study sites and setting

This investigation was performed in Jiangxi province, China, in a study site of the ISSC project. The site is termed County A in order to respect and guarantee the anonymity of the informants as requested by, and agreed with, the ethical authorities granting permission for this research. This study is based on data from County A, for which characteristics are presented in Table 1. Key information on the implementation of ISSC in County A is provided in Table 2.

**Table 1:** Basic characteristics of county A in 2011

Variables	Units
Area	2035 km^2^
Population	386,000
Annual income per capita of rural residents	7400 RMB^a^
No. of towns	21
No. of villages	182
No. of village clinics	206
No. of village doctors	579
Incidence of infectious disease^b^	668/10^5^/year

^a^Rinminbi; in 2012, the exchange of Chinese currency was about 6.31 RMB for 1 US$ according to the World Bank

^b^Reported by County A’s County Center for Disease Control and Prevention

**Table 2:** ISSC implementation in county A

	Phrase 1_Pilot study	Phrase 2_Formal study
Time period	August 1, 2011 - March 31,2012	April 1, 2012 - March 31, 2014
Sampling strategy	Purposive cluster sampling with town as the basic sample unit	Purposive cluster sampling with town as the basic sample unit
Towns involved	2	9 (including the 2 pilot towns)
Village clinics involved	19	75

### Data collection

We designed a questionnaire for the village clinics and a questionnaire for the village doctors in order to measure and value costs. The village clinic questionnaires collected information on the population served, outpatient volume, staff, computers, clinic incomes and expenditures, and the way in which the syndromic surveillance system had been implemented. Staff questionnaires collected individual information on the daily working time, and the time spent on the surveillance both before and after becoming familiar with the tasks involved in the surveillance.

This survey was part of a before-after study of an economic evaluation. It was performed using a non-randomized cluster sampling strategy following the ISSC project; the sampling units were towns. We approached 56 village clinics with the clinic questionnaire and one village doctor for each of the 56 village clinics with the staff questionnaire. The number of valid clinic and staff questionnaires received were, respectively, 51 and 53.

We also collected data on reported events by village clinics logged in an internal database of the ISSC project. Our hypothesis was that to report more events consumes more time in general. It was, therefore, considered meaningful to explore the daily time spent on the surveillance if the time was connected with the number of reported events on outpatients.

### Data analysis

#### Costs of time

Data from village clinics and staff questionnaires were first entered into Epidata 3.02 (the EpiData Association, Odense, Denmark), and then exported to Stata (StataCorp LP, Texas, USA) for descriptive analysis.

The average number of daily reported events of a village clinic for every 1000 of the population was the activity unit of this study. This unit (Y) was calculated as:

1 $$ \mathrm{Y} = \frac{\mathrm{R}\ast 1,000}{\mathrm{D}\ast \mathrm{P}} $$

Where R: number of reported events during a certain time period; D: number of days covered by the same time period; P: population covered.

The cost of time to report data for every 1000 of the population was the central measure of this study. Staff time costs were measured by multiplying the number of full time employment equivalents by their annual salaries and benefits, as suggested by several previous studies [9–12]. We used the clinic’s net income as a proxy for the village doctor’s salary and benefits since village doctors in China earn their living as a share of the gross income and expenditure of the village clinic. Costs of time (C) would be:

2 $$ \mathrm{C}=\frac{\mathrm{t}}{\mathrm{T}}\ast \mathrm{I} $$

Where t: daily staff time from a village clinic devoted to SSS for every 1000 of the population; T: total daily duty time of a reporter from a village clinic devoted to all services; I: net income of a village clinic for every 1000 of the population.

### Depreciation and opportunity costs of computers

We annualized computer purchasing costs according to their usage life and depreciation rate, as shown in equation (3).

3 $$ \mathrm{Equivalent}\ \mathrm{annual}\ \mathrm{cost} = \mathrm{the}\ \mathrm{capital}\ \mathrm{outlay}/\ \left[\mathrm{Annuity}\ \mathrm{factor},\ \mathrm{n}\ \mathrm{period},\ \mathrm{interest}\right] $$

Computer usage life in China is 6 years according to the WHO-CHOICE project [13]. We chose to use 5 % as the discount rate for the depreciation and opportunity costs of computers, as suggested, in order to be comparable with other studies on costs [14]. Consequently, the annuity factor for computers was 5.0757 [14].

We needed to allocate the annual depreciation and opportunity costs of computers to the syndromic surveillance project, since the computers were not solely used for the surveillance. The allocation basis was the usage percentage of the computers in each village clinic devoted to the surveillance.

Ethical approval for this study was obtained from Heidelberg University in Germany and Fudan University in People’s Republic of China.

## Results

### Characteristics of the investigated village clinics and village doctors

During the period of the survey, the total population covered by all 56 village clinics was 109,490, and the total number of village doctors was 123. Thirty-one village clinics (61 %) fully depended on one village doctor from each clinic to report data daily, whereas 20 clinics appointed additional village doctors to assist or substitute routine data reporters in their absence. Of those 53 reporters who were village doctors from village clinics, 49 were male. The population of a village, the number of village clinics at a village and the number of village doctors at a clinic are shown in Table 3 as well as the ages of village doctors.

**Table 3:** Characteristics of the investigated village clinics and village doctors in 2012

	Population of a village	No. of clinics at a village	No. of village doctors at a clinic	Age of village doctors
P25	1437	1	1	34
Median	1980	1	1	38
P75	2173	1	2	42

### Reported events and daily time on data collection from village clinics

Table 4 shows the numbers of outpatient visits and reported events from each of the village clinic to the ISSC from April 1 2012 to June 30 2013. The median number of daily reported events to the ISSC for every 1000 of the population was 3 (the 25 percentile (P25) = 2, the 75 percentile (P75) = 4).

**Table 4:** Number of outpatient visits and reported events to the ISSC from a village clinic from April 1 2012 to June 30 2013

	Total reported events per 1000 people	Total outpatient visits per 1000 people	Reported events/ outpatient visits (%)	Daily reported events per 1000 people	Daily outpatient visits per 1000 people
P25	931	2185	29.4	2	5
Median	1267	3375	42.3	3	7
P75	1932	4150	60.1	4	9

According to the village doctors, targeted syndromes were present in 42.3 % (P25 = 29.4, P75 = 60.1) of all out-patient visits and reported to the ISSC.

Daily time for data collection fell after 15 months’ implementation compared with the daily time for the first week of data collection, as stated by the majority (77.4 %) of the 53 data collectors. Table 5 presents the daily time needed for collecting data for the ISSC at each village clinic and as a percentage of the total duty time at the corresponding village clinics. Forty out of the 53 data collectors (77.5 %), who were village doctors from the village clinics, worked more than 480 min (P25 = 480, P75 = 720) per day as their regular duty time. Data collection for the ISSC for every 1000 of the population occupied a median of 4.2 % (2.1, 6.3) of a village doctor’s duty time. Forty out of the 52 data collectors (76.9 %), with missing data from one data collector, reported data for the ISSC after their duty working hours. According to our conversations with the village doctors during the survey, the reasons for working outside duty time for the surveillance were: 1) some preferred to enter data after work, either at village clinics or at their home; and 2) some had to enter data outside duty time because electricity or internet access were interrupted during working time, which happened from time to time in rural China.

**Table 5:** Costs of time of daily data collection and reporting for ISSC in 2012

	Reporting time in minutes per 1000 population (A)	Total time in minutes for all duties per 1000 population (B)	Percent of time in reporting per 1000 population (C)	Net income of a village clinic in 2012 in RMB (D)	Net income serving for 1000 population in RMB (E)	Costs of time in reporting for 1000 population in RMB (F)	Adjusted costs of time in reporting for 1,000 population in RMB (G)
P25	7	480	2.1	36,000	23,000	567	656
Median	12	600	4.2	52,500	27,579	1029	1250
P75	27	720	6.3	90,000	45,455	2000	3000

The exchange of Chinese currency was 6.31RMB for 1 US$ in 2012 according to the World Bank. We calculated percent of time in reporting, cost of time in reporting and the adjusted costs of time in reporting using the following formulas: C = A/B, F = E*C, G = F*150 %

Initial training was provided for the village doctors who were to collect the data. The time for the village doctors to attend initial training ranged from one day plus one hour to one and a half days. Other time consumed by the village doctors for the surveillance was the time they spent assisting staff from the County CDC in verifying signals. The time needed for signal verification by the village doctors was negligible, as shown in the following section.

### Warning signals detected based on reported events from village clinics

Eight signals were detected during the 15 months’ implementation period from the 56 village clinics. The number of signals per surveillance unit per day was close to zero. Five out of the eight signals were ruled out by the county CDC staff through telephone investigations, and the other three led to fieldwork investigation. Those investigation were performed by staff from the county CDC and assisted by village doctors from the three respective village clinics. Among those three, one signal was judged by the staff from the County CDC as not relevant to an outbreak, and the other two were suspected to be warnings of outbreaks. One of the two was confirmed as a new influenza A (H1NI) virus infection with laboratory tests. It was under control as the County CDC together with related health facilities and primary schools started to take coping measures as they began the investigation. The other signal was not confirmed with laboratory tests. The staff of the County CDC closely followed up the signal and no outbreaks occurred.

### Net incomes of village clinics and their village doctors

According to our survey, township hospitals or villages were the registered owners of village clinics in County A, whereas village doctors in County A enjoyed the benefits of ownership of those village clinics that they worked at. This means that the village doctors generated the raw incomes of the village clinics either by direct charges for medical treatments or medicines or by receiving payments from the government for providing certain services (e.g. performing the ten public health services included in the essential package in rural China). It also means that the village doctors needed to take care of the expenditures incurred in their clinics, which included the costs of medicines and medical supplies, the rents and maintenance of clinic buildings and their operation, and the transportation for medical or public health services. The net incomes of the village doctors came from the raw incomes of their clinics deducted by the expenditures incurred by their clinics.

Table 5 shows the distribution of net income per 1000 of the population of each village clinic. For every 1000 of the population, the median net income of a village clinic was 27,579 RMB (P25 = 23,000, P75 = 45,455).

### Costs of data collection by village doctors at village clinics for the ISSC

The surveillance itself was not supposed to be an after duty time job; however, the majority of village clinics reported data after routine working time, as reported in the former section. Working time after duty time should be valued more given that, according to the Labor Law of the People’s Republic of China [15], employers should pay their employees at least 150 % of salaries for after duty time work. We therefore adjusted the costs by multiplying them by 150 %, and presented the time costs of data collection both before and after the adjustment in Table 5. The adjusted median cost of staff time in 2012 was 1250 RMB (P25 = 656, P75 = 3000). As we reported in the former section, to perform the surveillance, village doctors also spent time on initial training and assistance in signal verification besides collecting data. This survey only focused on the costs of data collection.

The purchasing cost of a computer for the syndromic surveillance in county A in 2012 was 3400 RMB. Annual depreciation and opportunity cost of a computer in 2012 was therefore 670 RMB according to formula (5). We calculated the annual depreciation and opportunity costs of computers for the ISSC to be 134 RMB (P25 = 101, P75 = 335), with the median percentage of usage of computers for the ISSC 20 % (P25 = 8, P75 = 49).

The adjusted total cost of data collection at the village clinics for the surveillance, which included staff time costs, and the depreciation and opportunity costs of computers, was 1423 RMB (P25 = 857, P75 = 3284).

## Discussion

This survey represents a first step in exploring the operating cost of a syndromic surveillance system. It demonstrated a detailed process in calculating the costs of data collection at village clinics, including the cost of staff time, and the depreciation and opportunity cost of computers.

The average net income of a village resident in county A in 2012 was 8737 RMB [16]. Therefore, for village doctors or village clinics, the cost of collecting data for the syndromic surveillance system in County A for a population of 1000 in 2012 is 16 % (P25 = 10, P75 = 38) of the average net income of a resident in County A. It is 14 % (P25 = 8, P75 = 34) if we compared the cost of staff time in data collection with the net income of a resident in County A in 2012. According to these percentages, we consider the cost of data collection to be low. This conclusion is different from a study which found that implementing new data collection processes involved prohibitive costs [17].

We believe that the adjusted total costs of data collection at the village clinics for the syndromic surveillance were an appropriate proxy of the costs of the village clinics for participating in the syndromic surveillance services. Our reason was that the other costs incurred at the village clinics associated with participation in the syndromic surveillance included the time costs of the initial training and of the signal verification and investigations, and that these costs were negligible to the clinics because they were covered by the County CDC and researchers. Detailed reasons were: 1) the time for the initial training was minimal (1 day plus 1 h to half a day) considering that this surveillance was implemented as a daily routine; 2) the number of signals per surveillance unit per day was close to zero and fieldwork investigations were mainly performed by staff from the county CDC, although the village doctors whose reported events triggered the signals may assist the investigation.

The percentage of reported events among the total outpatient visits from other studies was 26.7 % [18] or 75 % [19] which spans the median percent found in this study. Further studies may need to explore this uncertainty regarding the percentages of reported events among total outpatient visits. Our reason was that these percentages relate to the workload in data collection for surveillance, even if their influence on the effectiveness of the surveillance is not yet known.

Understaffing was viewed as a challenge for public health surveillance [20]. Health care workers have repeatedly shown poor compliance with additional administrative tasks, such as data collection for surveillance [20]. In addition to studying the cost of data collection, information concerning the willingness and motivation of staff to undertake a syndromic surveillance system would be helpful for the policy makers.

The limitations of this study include: 1) it may under-estimate net incomes of village clinics and village doctors since income is a sensitive topic, accurate income data may be difficult to obtain [21]; consequently, this survey may over- or under-value village doctors’ time; 2) it is based on data from only one study site, and therefore caution must be given if generalizing for the rest of rural China.

## Conclusions

The total costs of collecting data by village doctors from village clinics in County A for the syndromic surveillance system were calculated to be low as compared with the average net income of a village resident in County A. The estimated cost of data collection was 1423 RMB in 2012 (P25 = 857, P75 = 3284), and this may provide a reference value for policy makers regarding a possible subsidy for village clinics to participate in similar syndromic surveillance systems.
